# Prognostic role of *EGFR* gene copy number and KRAS mutation in patients with locally advanced rectal cancer treated with preoperative chemoradiotherapy

**DOI:** 10.1038/sj.bjc.6605853

**Published:** 2010-09-14

**Authors:** C Bengala, S Bettelli, F Bertolini, G Sartori, A Fontana, N Malavasi, R Depenni, S Zironi, C Del Giovane, G Luppi, P F Conte

**Affiliations:** 1 Division of Medical Oncology, Department of Oncology, Hematology and Respiratory Disease, University Hospital, University of Modena and Reggio Emilia, Via del Pozzo, 71, Modena - 41100, Italy; 2 Institute of Pathology and Cell Biology, Department of Laboratories, Pathology and Forensic Medicine, University Hospital, University of Modena and Reggio Emilia, Via del Pozzo, 71, Modena - 41100, Italy

**Keywords:** rectal cancer, neoadjuvant chemoradiotherapy, EGFR, KRAS

## Abstract

**Background:**

Epidermal growth factor receptor (EGFR), evaluated by immunohistochemistry, has been shown to have prognostic significance in patients with colorectal cancer. Gene copy number (GCN) of EGFR and KRAS status predict response and outcome in patients treated with anti-EGFR therapy, but their prognostic significance in colorectal cancer patients is still unclear.

**Methods:**

We have retrospectively reviewed the baseline EGFR GCN, KRAS status and clinical outcome of 146 locally advanced rectal cancer (LARC) patients treated with preoperative chemoradiotherapy. Pathological response evaluated by Dworak's tumour regression grade (TRG), disease-free survival (DFS) and overall survival (OS) were analysed.

**Results:**

Tumour regression grade 4 and TRG3–4 were achieved in 14.4 and 30.8% of the patients respectively. Twenty-nine (19.9%) and 33 patients (19.2%) had an EGFR/nuclei ratio >2.9 and CEP7 polisomy >50% respectively; 28 patients (19.2%) had a KRAS mutation. Neither EGFR GCN nor KRAS status was statistically correlated to TRG. 5-year DFS and OS were 63.3 and 71.5%, respectively, and no significant relation with EGFR GCN or KRAS status was found.

**Conclusion:**

Our data show that EGFR GCN and KRAS status are not prognostic factors in LARC treated with preoperative chemoradiation.

Epidermal growth factor receptor (EGFR) protein expression, evaluated by immunohistochemistry (IHC), has been shown to have prognostic significance in patients with colon cancer (Watanabe *et al*, 2001). In locally advanced rectal cancer (LARC) treated with preoperative chemoradiotherapy (CTRT), baseline EGFR (IHC) expression predicts a poor tumour downstaging (Kim *et al*, 2006) and is also an independent prognostic factor for local recurrence (Azria *et al*, 2005; Giralt *et al*, 2005; Li *et al*, 2006). Moreover, we have shown that EGFR expression on the residual tumour after neoadjuvant CTRT is an independent prognostic parameter for disease-free survival (DFS; Bertolini *et al*, 2007). However, IHC is a semi-quantitative method that lacks a standardised immunostaining and scoring system and is subject to inter-observer variation (Atkins *et al*, 2004; Shia *et al*, 2005). *EGFR* gene copy number (GCN) determined by FISH has been proposed as a more reliable assay than IHC to determine the sensitivity of anti-EGFR drugs (Chung *et al*, 2005; Moroni *et al*, 2005; Cappuzzo *et al*, 2008) but the prognostic role of EGFR GCN is unclear.

KRAS mutation status has been shown to be a predictive factor for response to anti-EGFR monoclonal antibodies in metastatic colorectal cancer (Bokemeyer *et al*, 2009; Van Cutsem *et al*, 2009) and, recently, KRAS wild-type status has been recognised by the US FDA and EMEA as a predictive factor to select patients candidate to receive cetuximab or panitumumab. We have also shown that EGRF GCN and KRAS mutation status correlate with pathological response to preoperative chemoradiation and cetuximab in LARC (Bengala *et al*, 2009).

To further explore the prognostic and predictive role of EGFR GCN and KRAS mutation status, we have analysed our database of patients with LARC treated with preoperative radiation therapy and fluoropyrimidine-based chemotherapy. Objectives of the study were to correlate the GCN and KRAS status with the pathological response according to Dworak's tumour regression grade (TRG), DFS and overall survival (OS).

## Materials and methods

### Patients and treatment

We have retrospectively analysed data of 146 patients with LARC who were treated with preoperative chemotherapy at our institution between May 1998 and October 2005. The treatment included pelvic radiotherapy (2 Gy per fraction for 25 fractions) in a large pelvic field involving the tumour mass and regional lymph nodes, and concomitant chemotherapy with continuous infusion 5-fluorouracile (5-FU) with or without oxaliplatin or capecitabine. 5-Fluorouracil was administered at the dose of 225 mg m^−2^ per day as i.v. continuous infusion 7 days a week for 5 weeks; capecitabine was administered at 825 mg m^−2^ b.i.d. continuously for all the duration of radiotherapy; oxaliplatin was administered at 60 mg m^−2^ weekly in combination with 5-FU 225 mg m^−2^ per day as i.v. continuous infusion 7 days a week for 5 weeks. Surgery was performed 6–8 weeks after the end of combined treatment. A more detailed report of the staging procedure and treatment plan has been already published (Gavioli *et al*, 2005).

### Tissue samples and pathology assessment

All patients underwent tumour biopsy for diagnostic purpose before starting the treatment. Several 3 *μ*m-thick sections were obtained from a representative formalin-fixed and paraffin-embedded block where the neoplastic component was at least the 80% of the tissue. At the time of surgery, pathologic evaluation in the resection specimens included TNM categories, stage grouping, number of examined/involved lymph nodes and tumour differentiation. All pathological and molecular assessments were performed by dedicated pathologists at our University Hospital. Tumour regression was semi-quantitatively determined by the amount of viable tumour *vs* the amount of fibrosis, as described by Dworak *et al* (1997) and validated by Rodel *et al* (2005). Tumour regression grade 0 was defined as no regression; TRG1, minor regression (dominant tumour with fibrosis in ⩽25% of the tumour mass); TRG2, moderate regression (dominant tumour with fibrosis in 26–50% of the tumour mass); TRG3, good regression (more than 50% tumour regression) and TRG4, total regression (no viable tumour cells, only fibrotic mass).

### EGFR FISH

Fluorescence *in situ* hybridisation (FISH) studies were performed on selected sections of paraffin-embedded tissue areas, containing representative malignant cells, using the LSI EGFR Spectrum Orange/CEP7SpectrumGreen probe (Vysis Inc., Downer's Grove, IL, USA). Tissue sections of 4 *μ*m thickness were placed on electrostatically charged slides, air dried and baked overnight at 56°C. The slides were de-waxed in xylene for 2 × 15 min, immersed in 100% ethanol for 2 × 5 min and in 95% ethanol for 2 × 5 min. Air-dried tissue sections were treated with a Paraffin Pretreatment Kit (Vysis Inc.). The slides were briefly incubated in 0.2 mol l^−1^ HCl for 20 min, washed with Wash Buffer, incubated for 30 min at 80°C with Pretreatment Solution (NaSCN), washed with Wash Buffer and finally treated in a Protease I solution (0.5 mg ml^−1^ protease buffer; pH 2) for 10–12 min at 37°C.

After adding 10 *μ*l of the hybridisation probe and placing a coverslip, denaturation and hybridisation of DNA was performed using the metal block of a thermocycler (HyBrite; Vysis Inc.). The denaturation was carried out at 83°C for 3 min and the hybridisation was carried out overnight at 37°C. After hybridisation the excess of the probes was washed in 2 × SSC/0.3% NP-40 at 73°C for 2 min. The nuclei were counterstained with 1000 ng ml^−1^ DAPI/Antifade (4.6-diamidine-2-phenyl indole; Vysis Inc.). For the scoring, a Zeiss Axioscope fluorescence microscope (Carl Zeiss Inc., Jena, Germany) was used, equipped with a specially designed filter combination: the EGFR sequence was visualised with a Orange filter, the chromosome 7 centromere sequence was visualised with a Green filter and the nuclei were identified with a DAPI filter. A triple band pass filter (Orange, Green and DAPI; Vysis Inc.) was also used. Hybridisation signals were scored in at least 200 intact non-overlapping nuclei and FISH analysis was performed independently by two observers using constant adjustment of microscope focus because signals were located at different focal planes. Representative images of each specimen were acquired with a high-performance CCD camera in monochromatic layers that were subsequently merged by the Quips PathVysion Software (Vysis Inc.).

Epidermal growth factor receptor gene status was scored as the average number of EGFR red signals per nucleus and as the ratio between EGFR red signals and CEP7 green signals. Polisomy of *EGFR* gene was defined as an increase of EGFR red signals (⩾ three signals per nucleus) paralleled by the same increase of chromosomes 7 (where the *EGFR* gene is located) as measured by the number of CEP 7 green signals per nucleus. High GCN was defined as EGFR/nuclei ratio ⩾2.9 or an EGFR/CEP7 polysomy >3 in at least 50% of the cells. All cases were scored and reviewed by two observers (SB and NB) and inter-observer disagreement was discussed in an institutional meeting.

### DNA extraction and KRAS mutation analysis

KRAS mutation status was analysed at the Laboratory of Cell Biology of the Department of Pathology, University of Modena and Reggio Emilia, Modena, Italy.

Three haemaxytolin-eosin-stained sections (5 *μ*m thick) from a representative paraffin-embedded block were applied on non-cover-slipped slides for microdissection and DNA extraction. Briefly, microdissection was performed under direct observation with an inverted microscope using a sterile needle. Each microdissected sample was directly transferred to an Eppendorf tube containing digestion buffer (2 mg ml^−1^ proteinase K in 50 mM Tris (pH 8.5), 1 mM EDTA, 0.5% Tween 20). The tubes were then incubated overnight at 56°C, and followed by 10 min of incubation at 95°C to eliminate any remaining proteinase K activity. PCR was performed in 20 *μ*l reactions containing 2.0 *μ*l DNA, 2 *μ*l commercial PCR buffer (Applied Biosystems, Foster City, CA, USA), 2.0 mM of MgCl_2_, 200 mM of each dNTP, 20 pmol of each primer, and 3 U AmpliTaq Gold polymerase (Applied Biosystems). PCR reaction was carried out on Uno II Thermoblock (Biometra, Gottingen, Germany). Initial denaturation at 95°C for 10 min was followed by 41 cycles, and a final extension step (10 min at 72°C). The cycles included denaturation at 95°C for 1 min, annealing at 52°C for 1 min and extension at 72°C for 2 min. Exon 2 of KRAS was PCR amplified using intron-based primers to investigate the mutational status of KRAS codons 12 and 13, because it is frequently founded mutated in colorectal cancer. The forward and reverse oligonucleotide primers used to amplify KRAS exon 2 were: forward, 5′-CATGTTCTAATATAGTCACA-3′ reverse, 5′-AACAAGATTTACCTCTATTG-3′.

The amplified DNA was electrophoresed on 2% agarose gel for 1 h at 110 V. The amplification products were then purified by using MinElute PCR purification Kit (Qiagen, Valencia, CA, USA) as indicated by the manufacturer. PCR products were then amplified in both directions with ABI Prism BigDye Terminator version 1.1 Cycle Sequencing kit (Applied Biosystems), using the same primers as those used for PCR. PCR products were finally purified by Centri-Sep Spin Columns (Applied Biosystems) and subsequently ran on the ABI Prism 310 Automatic Sequencer (Applied Biosystems). The data were analysed with the Sequencing Analysis 5.2 software (Applied Biosystems).

### Statistical analysis

The study, including the molecular analysis, was approved by the ethical committee of the Province of Modena.

Tumour downstaging and the pathological response, based on Dworak's TRG, were associated with biological characteristics with Fisher's exact test.

Disease-free survival was defined as the interval from the date of diagnosis until the date of tumour recurrence, second primary tumour or death with or without recurrence. Overall survival was defined as the date of study entry until the date of death. The DFS and OS curves were calculated according to the Kaplan–Meier method. Long-rank test was used to compare DSF and OS according to pathological response and tumour biomarkers.

Hazard ratios and 95% confidence intervals for recurrence or death were calculated to evaluate the association with the pathological response and the tumour biomarkers using the univariate Cox proportional hazard model.

Statistical significance was set at *P*<0.05. All the analyses were conducted using STATA 10.0 (College Station, TX, USA).

## Results

Patient characteristics are described in Table 1. Median age was 64 years (range 26–78). At diagnosis, clinical stage defined with ultrasonography was stage II in 59 patients (IIA 48 patients, IIB 11 patients), stage III in 83 patients (IIIA 5 patients, IIIB 78 patients) and stage IV in 4 patients. All the patients received pelvic radiation therapy; concomitant chemotherapy was 5-FU in 98 patients (67.1%), 5-FU in combination with oxaliplatin in 34 patients (23.3%) and capecitabine in 14 patients (9.6%). A pathological complete response classified as Dworak's TRG4 was achieved in 21 patients (14.4%); a TRG3 was observed in 24 patients (16.4%), TRG2 in 46 patients (31.5%); TRG1 in 48 patients (32.9%), TRG0 in 3 patients (2.1%). Tumour and lymph node downstaging was observed in 85 patients (58.2%).

### Biomarker expression and response

EGFR GCN was evaluated as EGFR/nuclei ratio and as CEP7 polisomy. Median EGFR/nuclei ratio was 2.3 (range 1.1–3.5). Median CEP7 polisomy was 28.3% (range 0–78%). A high EGFR/nuclei ratio (⩾2.9) and a low EGFR/nuclei ratio (<2.9) was present in 19.9 and 78.8% patients respectively; a high CEP7 polisomy (⩾50%) and a low polisomy (<50%) was observed in 22.6 and 75.3% patients respectively. Among the 113 assessable patients with low EGFR/nuclei ratio, 15 (13.3%) and 36 (31.3%) had a TRG4 and TRG3–4 respectively, compared with 6 (22.2%) and 9 (33.3%) of the 27 assessable patients with high EGFR/nuclei ratio (*P*=0.19 and 0.53). Moreover, 14 (12.7%) and 36 (32.7%) of the 110 assessable patients with low CEP7 polisomy had a TRG4 and TRG3–4 respectively, compared with 7 (23.3%) and 9 (30.0%) of the 30 patients with high CEP7 polisomy (*P*=0.13 and 0.48) (Table 2).

The downstaging rate was 59.6 *vs* 65.5% in patients with low and high EGFR/nuclei ratio respectively (*P*=0.36) and 64.8 and 48.5% in patients with low and high CEP7 polisomy respectively (*P*=0.07) (Table 2).

A wild-type KRAS was reported in 79.5% of the patients and in these patients the rate of TRG4 was 16.7% a mutated KRAS was found in 19.2% of the patients with a TRG4 of 7.4% this difference is not significant (*P*=0.18). The rate of TRG3–4 was higher in wild-type KRAS (35.1%) in comparison to mutated status (18.5%) and this difference approaches a statistical significance (*P*=0.07) (Table 2).

### Biomarker expression and survival

**Figure 1:**
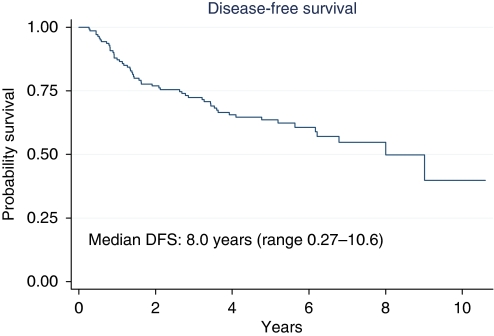
Disease-free survival of entire group of patients.

**Figure 2:**
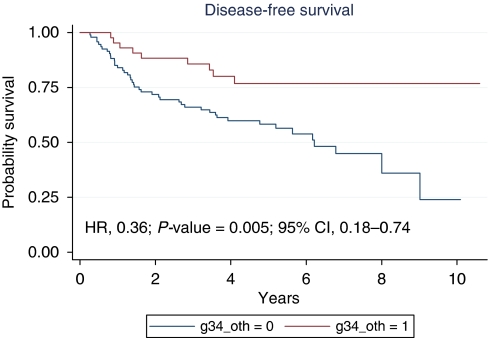
Disease-free survival according to the tumour regression grade.

At a median follow-up of 4.45 years (range 0.29–10.6), median DFS was 8 years (range 0.27–10.6) (Figure 1). The 5- and 10-year DFS were 63.6 and 39.8% respectively. The 5-year DFS was 77% for the patients with TRG3–4 and 58% for the patients with TRG0–2 (HR, 0.36; 95% CI: 0.18–0.74; *P*=0.005; Figure 2). The 5-year DFS was 64.2 and 62.2% for patients with low *vs* high EGFR/nuclei respectively (HR, 0.99; 95% CI: 0.51–1.94; *P*=0.99); 67.2 and 51.1% for patients with low *vs* high CEP7 polisomy respectively (HR, 1.43; 95% CI: 0.78–2.60; *P*=0.24); 64.7 and 61.3% for patients with wild-type *vs* mutated KRAS respectively (HR, 0.94; 95% CI: 0.49–1.83; *P*=0.86).

**Figure 3:**
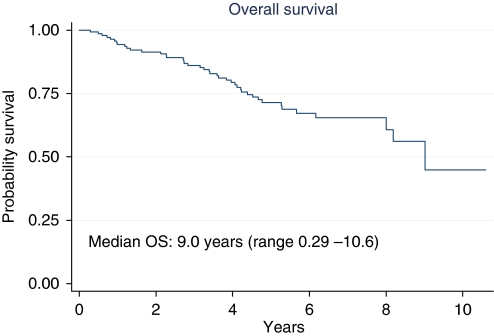
Overall survival of the entire group of patients.

**Figure 4:**
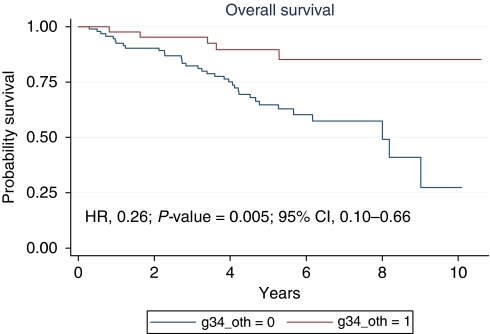
Overall survival according to the tumour regression grade.

Median OS was 9.0 years (range 0.26–10.6) (Figure 3). At 5 and 10 years OS rates were 71.5 and 44.9% respectively. At 5 years, OS rates were 89.7 and 64.8% for the patients who achieved a TRG3–4 and patients with TRG 0–2 respectively (HR, 0.26; 95% CI: 0.10–0.66; *P*=0.005) (Figure 4); 72.3% for both wild-type and mutated KRAS patients; 71.0 and 76.2% for patients with low *vs* high EGFR/nuclei ratio respectively (HR, 1.13; 95% CI: 0.54–2.39; *P*=0.74); 75.3 and 60.9% for the patients with low *vs* high CEP7 polisomy respectively (HR, 1.83; 95% CI: 0.94–3.57; *P*=0.07).

## Discussion

Epidermal growth factor receptor expression has been shown to be associated with disease recurrence and poor survival in colon cancer (Iqbal and Lenz, 2001; Watanabe *et al*, 2001; Galizia *et al*, 2006). Moreover, the predictive role of EGFR expression on tumour response and locoregional recurrence has been extensively investigated in patients with LARC treated with preoperative chemoradiation therapy (Giralt *et al*, 2002; Azria *et al*, 2005). All the data show that EGFR expression, evaluated by IHC, is a predictive factor for poor tumour response and local recurrence after preoperative chemoradiation therapy and curative surgery for rectal cancer. Moreover Giralt *et al* (2005) and Li *et al* (2006) reported that baseline EGFR expression is an independent prognostic factor for DFS and distant metastasis-free survival. We recently analysed patients with LARC treated with preoperative CTRT and we were unable to confirm that baseline EGFR expression evaluated by IHC is a predictive factor for response as well as prognostic factor on survival; on the contrary, EGFR expression by IHC on residual tumour after preoperative chemoradiation is an independent poor prognostic factor for disease recurrence (Bertolini *et al*, 2007). Possible explanation for these discrepancies can be unreliable techniques of immunostaining and scoring, heterogeneity of EGFR expression, and high and low affinity of EGFR. Moreover polymorphism of EGFR has been shown to predict tumour response to CTRT and locoregional tumour recurrence after CTRT (Zhang *et al*, 2005; Spindler *et al*, 2006).

In this study we show that EGFR GCN is not predictive of response to preoperative chemoradiation therapy and is not a prognostic factor for DFS and OS. Interestingly, we had previously reported that high EGFR GCN is predictive of TRG3–4 in patients treated with chemoradiation in combination with cetuximab (Bengala *et al*, 2009). These evidences are not in contrast because while EGFR GCN may not have a prognostic value it can be a possible predictive factor of response to anti-EGFR monoclonal antibody cetuximab.

KRAS mutation status has been extensively studied as a predictive factor of resistance to anti-EGFR monoclonal antibodies. In our study, KRAS mutation status is not a predictive factor for neither pathologic response nor downstaging, although the rate of pathological regression grade 3–4 was 35.1 *vs* 18.5% in patients with wild-type and mutated KRAS status respectively (*P*=0.07). Moreover, KRAS status did not predict DFS and OS.

Two large prospective studies conducted in stage II–III colon cancer (Roth *et al*, 2010) and in advanced colorectal cancer (Bokemeyer *et al*, 2009; Van Cutsem *et al*, 2009) have shown that KRAS mutation is not a prognostic factor. Conversely, two large collaborative studies, the RASCAL trials, have reported an increased risk of recurrence and death in patients with colon cancer and KRAS mutation (Andreyev *et al*, 1998, 2001). In the study by Roth *et al* (2010), tumour specimens were prospectively collected and analysed in a central laboratory, whereas in the RASCAL trials the specimens were retrospectively analysed in local laboratories and this could explain the contradictory results. Moreover, the second RASCAL trial reported a poor outcome only for a small subset of patients bearing a G12V mutation raising the question on the possible prognostic relevance of specific KRAS mutations (Andreyev *et al*, 2001). Unfortunately the study by Roth *et al* did not have a sufficient statistical power to detect a possible prognostic role of different KRAS mutations. Differences in patient population (colorectal *vs* locally advanced rectal), treatments (chemotherapy *vs* chemoradiation) and sample sizes might account for the inconsistency of available data on prognostic role of KRAS mutation across these studies.

In conclusion, our data show that EGFR GCN and KRAS mutation status are neither predictive nor prognostic factors for pathological tumour response and DFS in LARC patients treated with preoperative chemoradiation. On the basis of these data, KRAS status should not be used to select therapies other than anti EGFR antibodies.

## Figures and Tables

**Table 1:** Patient characteristics

**Number of patients**	**146**	
**Median age, years (range)**	**64 (26–78)**	
**Characteristic**	***N***	**%**
*Gender*		
Male	86	58.9
Female	60	48.1
PS (ECOG scale)	0	100
		
*Clinical stage*		
uT2N1	5	3.4
uT3N0	48	32.9
uT3N1	63	43.2
uT4N0	11	7.5
uT4N1	15	10.3
Any TNM1	4	2.7
		
*Chemotherapy concurrent to RT*		
5-FU	98	67.1
5-FU+Oxaliplatin	34	23.3
Capecitabine	14	9.6
		
*EGFR/nuclei ratio*		
<2.9	115	78.8
⩾2.9	29	19.9
NA	2	1.4
		
*CEP7 polisomy (*>*3 copies)*		
<50%	110	75.3
⩾50%	33	22.6
NA	3	2.1
		
*KRAS*		
Wild type	116	79.5
Mutated	28	19.2
NA	2	1.4
		
*Pathological response (Dworak's grade)*		
TRG 4	21	14.4
TRG 3	24	16.4
TRG 2	46	31.5
TRG 1	48	32.9
TRG 0	3	2.1
NA	4	2.7
		
*Downstaging*		
Yes	85	58.2
Not	55	37.7
NA	6	4.1

Abbreviations: ECOG= eastern cooperative oncology group; EGFR=epidermal growth factor receptor; 5-FU=5-fluorouracile; NA= not available; PS=performance status; TRG=tumour regression grade.

**Table 2:** *EGFR* gene copy number, KRAS status, pathological response and downstaging

	**EGFR/nuclei ratio**			**CEP7 polisomy**			**KRAS status**		
	**<2.9**	**⩾2.9**	***P*-value**	**<50%**	**⩾50%**	***P*-value**	**Wild type**	**Mutated**	***P*-value**
*Pathological response*									
Assessable patients, *n*	113	27		110	30		114	27	
TRG 4, *n* (%)	15 (13.3)	6 (22.2)	0.19	14 (12.7)	7 (23.3)	0.13	19 (16.7)	2 (7.4)	0.18
TRG 3–4, *n* (%)	36 (31.9)	9 (33.3)	0.53	36 (32.7)	9 (30.0)	0.48	40 (35.1)	51 (8.5)	0.07
									
*Downstaging*									
Assessable patients, *n*	109	29		105	33		111	28	
Downstaging, *n* (%)	65 (59.6)	19 (65.5)	0.36	68 (64.8)	16 (48.5)	0.07	69 (62.2)	16 (57.1)	0.39

Abbreviations: EGFR=epidermal growth factor receptor; TRG=tumour regression grade.
